# Interaction of transcription factor FoxO3 with histone acetyltransferase complex subunit TRRAP modulates gene expression and apoptosis

**DOI:** 10.1016/j.jbc.2022.101714

**Published:** 2022-02-11

**Authors:** Lorenza Fusi, Rupesh Paudel, Katharina Meder, Andreas Schlosser, David Schrama, Matthias Goebeler, Marc Schmidt

**Affiliations:** 1 Department of Dermatology, Venereology and Allergology, University Hospital Würzburg, Würzburg, Germany; 2 Rudolf Virchow Center, Center for Integrative and Translational Bioimaging, University of Würzburg, Würzburg, Germany

**Keywords:** apoptosis, cell cycle, endothelial cell, FoxO, protein–protein interaction, transcription regulation, 4-OHT, 4-hydroxytamoxifen, ANGPT2, angiopoietin-2, BCL2L11 (also known as BIM), B-cell lymphoma 2–like protein 11, DB, DNA-binding domain, DBE, DAF-16 binding element, EC, endothelial cell, ER, estrogen receptor, FoxO, forkhead box subclass O, FRE, forkhead-responsive element, HA, human influenza hemagglutinin, HAT, histone acetyltransferase, HEK293, human embryonic kidney 293 cell line, HUVEC, human umbilical vein endothelial cell, IGFBP1, insulin-like growth factor–binding protein 1, IP, immunoprecipitation, LFQ, label-free quantification, MS, mass spectrometry, p27^kip1^, cyclin-dependent kinase inhibitor 1B, PI, propidium iodide, PIKK, PI3K-related kinase, PKB, protein kinase B, siSCR, scrambled siRNA, siTRRAP, TRRAP-targeting siRNA, TA, transactivation domain, Tip60, Lysine acetyl transferase 5 (also known as TAT interacting protein 60 kDa), TRRAP, transformation/transcription domain–associated protein

## Abstract

Forkhead box O (FoxO) transcription factors are conserved proteins involved in the regulation of life span and age-related diseases, such as diabetes and cancer. Stress stimuli or growth factor deprivation promotes nuclear localization and activation of FoxO proteins, which—depending on the cellular context—can lead to cell cycle arrest or apoptosis. In endothelial cells (ECs), they further regulate angiogenesis and may promote inflammation and vessel destabilization implicating a role of FoxOs in vascular diseases. In several cancers, FoxOs exert a tumor-suppressive function by regulating proliferation and survival. We and others have previously shown that FoxOs can regulate these processes *via* two different mechanisms: by direct binding to forkhead-responsive elements at the promoter of target genes or by a poorly understood alternative process that does not require direct DNA binding and regulates key targets in primary human ECs. Here, we performed an interaction study in ECs to identify new nuclear FoxO3 interaction partners that might contribute to FoxO-dependent gene regulation. Mass spectrometry analysis of FoxO3-interacting proteins revealed transformation/transcription domain–associated protein (TRRAP), a member of multiple histone acetyltransferase complexes, as a novel binding partner of FoxO family proteins. We demonstrate that TRRAP is required to support FoxO3 transactivation and FoxO3-dependent G1 arrest and apoptosis in ECs *via* transcriptional activation of the cyclin-dependent kinase inhibitor p27^kip1^ and the proapoptotic B-cell lymphoma 2 family member, BIM. Moreover, FoxO–TRRAP interaction could explain FoxO-induced alternative gene regulation *via* TRRAP-dependent recruitment to target promoters lacking forkhead-responsive element sequences.

The forkhead box O (FoxO) transcription factor subfamily is a part of the larger heterogeneous forkhead box (Fox) family of transcription factors (1). FoxO proteins share a highly conserved DNA-binding domain (DB), which recognizes two specific DNA sequences at the promoter of their target genes; the DAF-16 binding element (DBE) and the insulin-responsive element, together referred to as forkhead-responsive elements (FREs) (2). The eponymous factor for the former is DAF-16, the sole FoxO factor in *Caenorhabditis elegans*. In the worm, DAF-16 regulates longevity and quiescence under reduced insulin/insulin-like growth factor 1 signaling, for example, in response to nutrient deprivation (3, 4). In humans, there are three functional DAF-16 orthologs (in the following referred to as “FoxOs”), which are widely expressed and show redundant activities: FoxO1 (alternatively known as forkhead in rhabdomyosarcoma), FoxO3 (also known as forkhead in rhabdomyosarcoma-like 1), and FoxO4 (also known as acute lymphocytic leukemia 1-fused gene from X-chromosome) (2, 5). In addition, a fourth FoxO isoform, FoxO6, is present in mammals, which exhibits a more restricted tissue expression and different regulation and function (6). Similar to *C. elegans*, human FoxOs mediate responses to stress, such as growth factor deficiency, hyperglycemia, or accumulation of reactive oxygen species. Exposure to these stress stimuli promotes FoxO activity influencing cellular homeostasis, survival, and proliferation in a highly cell-specific and context-specific fashion (7).

Activity of FoxOs is strongly dependent on activation of the growth factor–induced and proto-oncogenic PI3K/protein kinase B (PKB, also known as AKT) pathway, which keeps FoxOs inactive by PKB-mediated phosphorylation of three specific amino acid residues (T32, S253, and S315 in FoxO3) leading to their nuclear exclusion and cytoplasmic retention (5, 8). Mutation of the three PKB phosphorylation sites to alanine (FoxO.A3) renders FoxOs insensitive to PKB regulation resulting in their constitutive nuclear localization and activation (5, 9, 10). Consequently, expression of those FoxO.A3 mutants is able to antagonize many PI3K/PKB-dependent functions, including their antiapoptotic and proliferative effects in tumor cells (10, 11) suggesting FoxOs to act as tumor suppressors downstream of PI3K/PKB signaling. Indeed, it was broadly demonstrated that forced FoxO expression or expression of FoxO.A3 variants, depending on the cellular context, induces cell cycle arrest or apoptosis by controlling expression of several cell cycle–relevant or apoptosis-relevant genes (2, 12). For instance, FoxO activation was found to block G1–S progression by promoting cyclin-dependent kinase inhibitor 1B (also known as p27^kip1^) expression (10, 11) and suppressing D-type cyclins (13, 14) in a wide variety of cells. These include not only primary cells but also cancer cell lines that exhibit constitutive PI3K/PKB activity because of inactivating mutations in the tumor suppressor phosphatase and tensin homolog. In a limited number of cell types, including lymphocytes and endothelial cells (ECs), FoxO3 activation also can induce apoptosis *via* transcriptional upregulation of proapototic genes, such as B-cell lymphoma 2–like protein 11 (BCL2L11, also known as BIM) (15, 16, 17). Intriguingly, Paik *et al.* (18) revealed that broad somatic deletion of all alleles of FoxO1, FoxO3, and FoxO4 initiated cell transformation in a highly cell type–specific manner producing mainly thymic lymphomas and EC-derived hemangiomas. These findings support the classification of FoxOs as context-specific tumor suppressors, but the exact mechanism underlying this context specificity is unknown. It is further unclear why some cells undergo a G1 cell cycle arrest followed by quiescence upon FoxO activation, whereas others such as ECs are prone to induce apoptosis.

In addition to their broad functional redundancy in the regulation of proliferation and apoptosis, recent knockout studies further revealed some tissue-specific functions of the different FoxO members. Germline disruption of FoxO1 in mice, for instance, leads to premature death because of defects in angiogenesis and vascular development, which were attributed to endothelial dysfunction (19, 20). Consistently, FoxO1 and FoxO3 were shown to play a central role in EC migration and vessel formation in adult mice (21) and this was proposed to depend on the ability of FoxO1 to link EC proliferation and metabolism (22).

We have previously investigated the endothelial transcriptome induced by a FoxO3.A3 mutant fused to the hormone-binding domain of the murine estrogen receptor (ER) (FoxO3.A3.ER) (15), which can be conditionally activated by addition of 4-hydroxytamoxifen (4-OHT) to the medium (16). Consistent with an earlier study in phosphatase and tensin homolog–negative renal carcinoma cells (13), our preceding study revealed that FoxOs can activate gene expression both directly by binding to FREs present in its target promoters and by an ill-defined indirect mechanism that does not require direct FRE binding (in the following referred to as “alternative” gene regulation). Intriguingly, we observed that in particular FoxO-induced apoptosis, the major outcome of FoxO activation in ECs at the cellular level (15, 23) was dependent on alternative gene expression as we identified BIM as essential alternatively regulated proapoptotic gene in primary human ECs (15). In addition, several established universal FoxO target genes, such as insulin-like growth factor–binding protein 1 (IGFBP1) (13) and p27^kip1^ (10) as well as a variety of cell type–specific FoxO targets, such as angiopoietin-2 (ANGPT2) (23) were directly regulated by conditional FoxO3 activation (15) suggesting that FRE-dependent gene expression is also subject to cell type–specific or context-specific modulation.

Beyond PKB-mediated phosphorylation, several other post-translational mechanisms have been described that can modulate FoxO activity and may explain alternative, tissue-, or context-specific gene expression controlled by FoxOs. These include not only activating and inactivating phosphorylation at PKB-independent sites, acetylation, methylation, or ubiquitination of FoxOs themselves but also regulation by interacting proteins (2, 5).

Here, we analyzed the nuclear interactome of FoxO3 in primary human ECs. We identified transformation/transcription domain–associated protein (TRRAP) as a new FoxO-binding protein conserved in different cell types and reveal TRRAP as an essential regulator of FoxO3-dependent classical and alternative gene regulation in ECs controlling essential FoxO responses such as cell cycle arrest and apoptosis.

## Results

### TRRAP is a novel interaction partner of FoxO transcription factors

**Figure 1:**
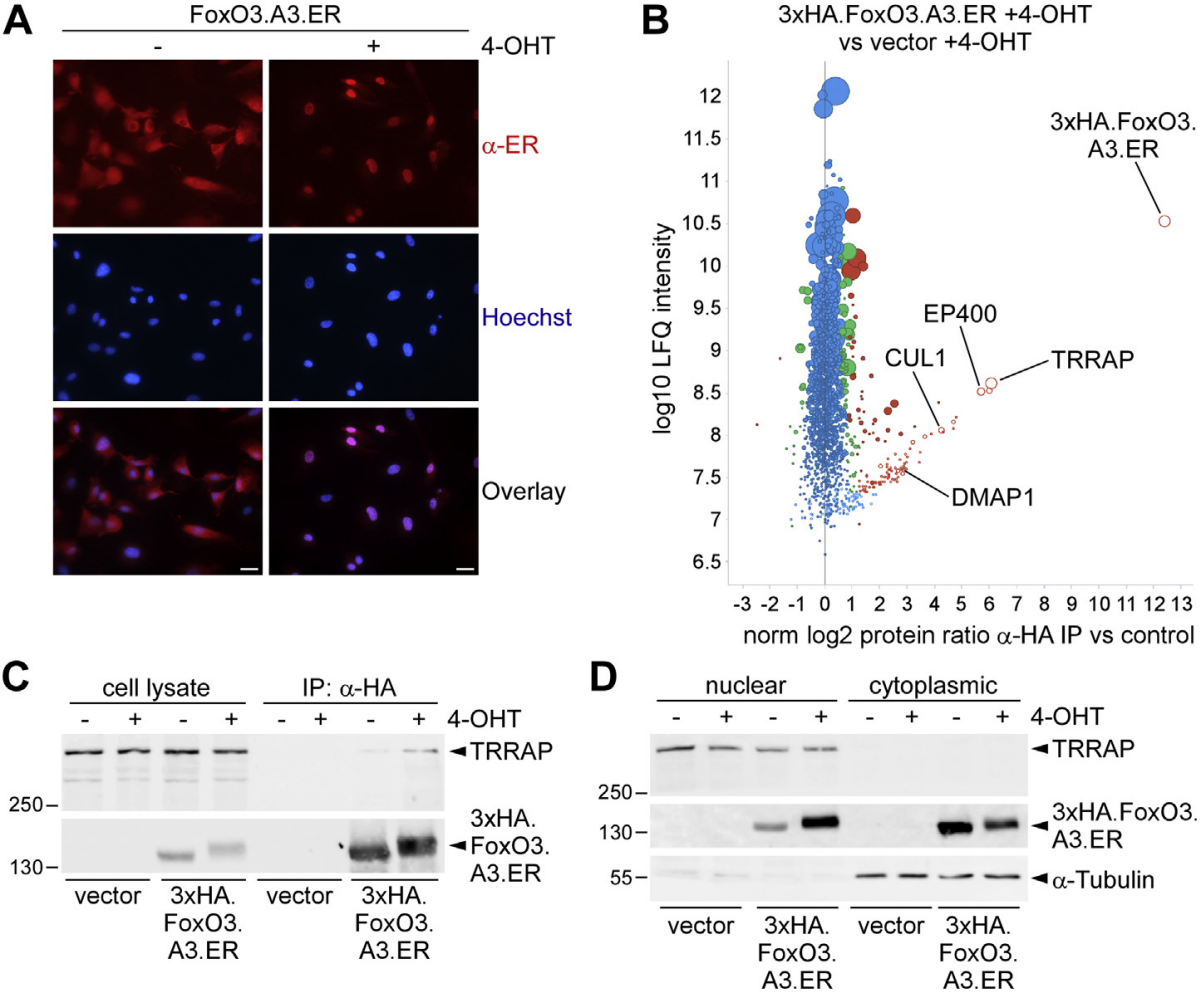
**TRRAP is a FoxO3-interaction partner in endothelial cells (ECs).***A*, α-ER immunofluorescence staining was performed to display localization of exogenous FoxO3.A3.ER 16 h after stimulation with or without 4-OHT in HUVEC infected with a FoxO3.A3.ER-encoding retrovirus. *Red channel*, α-ER antibody; *blue channel*, Hoechst nuclear staining. To facilitate interpretation, additional overlays of both channels are shown. The scale bar represents 25 μm. *B*, scatter plot showing the results of one 3xHA.FoxO3.A3.ER interactome analysis. The plot shows quantitative comparisons between the coimmunoprecipitated proteins detected in lysates of the 4-OHT-treated 3xHA.FoxO3.A3.ER-infected condition and those present in the 4-OHT-treated empty vector control. Lysates were taken 16 h after 4-OHT treatment to ensure nuclear localization without obvious apoptosis induction, as confirmed by parallel DNA profiling analysis (not shown, compare Fig. S3). *Red color marks*, proteins with significance of two; *green color marks*, proteins with significance of one; and *blue color marks*, nonspecifically bound proteins (for details, see Experimental procedures section). *Dot size* corresponds to the number of identified razor and unique peptides of the corresponding protein. *Open circles* denote the absence of a quantitative value in the empty vector–infected condition; missing values have been imputed (for details, see Experimental procedures section). Known TRRAP-interacting proteins present among the list of the top 30 enriched FoxO3 interaction partners are annotated. *C*, immunoblot, showing expression of the indicated proteins in total lysate or after immunoprecipitation with α-HA antibody from HUVECs infected with 3xHA.FoxO3.A3.ER or vector and stimulated with medium (−) or 4-OHT (+) for 16 h. *D*, Western blot analysis of nuclear and cytoplasmic extracts of 3xHA.HA.FoxO3.A3.ER-infected or empty vector–infected HUVECs incubated with (+) or without (−) 4-OHT for 16 h. α-Tubulin was chosen as cytoplasmic marker to demonstrate efficient cell fractionation. 4-OHT, 4-hydroxytamoxifen; ER, estrogen receptor; FoxO3, forkhead box subclass O3; HA, human influenza hemaglutinnin; HUVEC, human umbilical vein endothelial cell; TRRAP, transformation/transcription domain–associated protein.

To better understand FoxO-dependent gene regulation in ECs, we performed a mass spectrometric analysis of FoxO3-binding proteins in primary human umbilical vein endothelial cells (HUVECs). To specifically identify relevant nuclear regulators of FoxO3, we retrovirally expressed either an empty expression vector or a 3xHA (human influenza hemagglutinin [HA])-tagged FoxO3.A3.ER mutant (3xHA.FoxO3.A3.ER), through which nuclear localization can be induced conditionally within 16 h by 4-OHT treatment (Fig. 1*A*). Subsequently, we performed α-HA immunoprecipitations (IPs) with equal amounts of cell lysates of 4-OHT-treated 3xHA.FoxO3.A3.ER–infected or vector-infected cells using magnetic beads coupled to an α-HA antibody. The tryptic digest of the α-HA copurified proteins in both conditions was then analyzed by nano-liquid chromatography–tandem mass spectrometry (MS) to identify specific FoxO3-interacting proteins. This analysis revealed TRRAP as top enriched interaction partner of FoxO3 (Fig. 1*B*; see Table S1 for the comprehensive list of identified interaction partners with the top 30 enriched proteins highlighted in *light yellow*). TRRAP is a large pseudokinase belonging to the family of PI3K-related kinases (PIKKs), which regulate responses to stress, including DNA damage, genotoxic stress, and nutrient stress (24). Because TRRAP has an important function in transcription regulation by interacting with various transcriptional activation complexes (25), it qualified as a good candidate modulator of FoxO3 activity. Intriguingly, we in addition identified several known TRRAP-associated proteins as new FoxO3-interaction partners among the top 30 enriched binding proteins in our MS analysis (marked in Fig. 1*B* and Table S1) suggesting that FoxO3 also associates with other components of the TRRAP complex.

To confirm interaction of TRRAP to FoxO3, we performed independent co-IP experiments. α-HA IPs conducted with lysates of 3xHA.FoxO3.A3.ER–infected or empty vector–infected HUVECs revealed a prominent interaction of endogenous TRRAP with 3xHA.FoxO3.A3.ER in the presence of 4-OHT (Fig. 1*C*). In addition, we observed a weak interaction of endogenous TRRAP and 3xHA.FoxO3.A3.ER in α-HA IPs of mock-treated HUVECs expressing 3xHA.FoxO3.A3.ER. This was not because of unspecific binding of TRRAP to the used α-HA beads, as we failed to detect any TRRAP in α-HA IPs of vector-infected cells (Fig. 1*C*). We further could exclude a cytoplasmic interaction between 3xHA.FoxO3.A3.ER and TRRAP, as endogenous TRRAP was exclusively found in the nuclear fraction when nuclear and cytoplasmic proteins were separated by specific fractionation (Fig. 1*D*). By contrast, exogenous 3xHA.FoxO3.A3.ER showed a markedly increased nuclear localization upon 4-OHT treatment (Fig. 1*D*). Analogous to our immunofluorescence staining (Fig. 1*A*), however, we also detected a small fraction of 3xHA.FoxO3.A3.ER in the nuclear extracts of diluent-treated cells (Fig. 1*D*), implying that the interaction of both proteins took place in the nuclear compartment.

**Figure 2:**
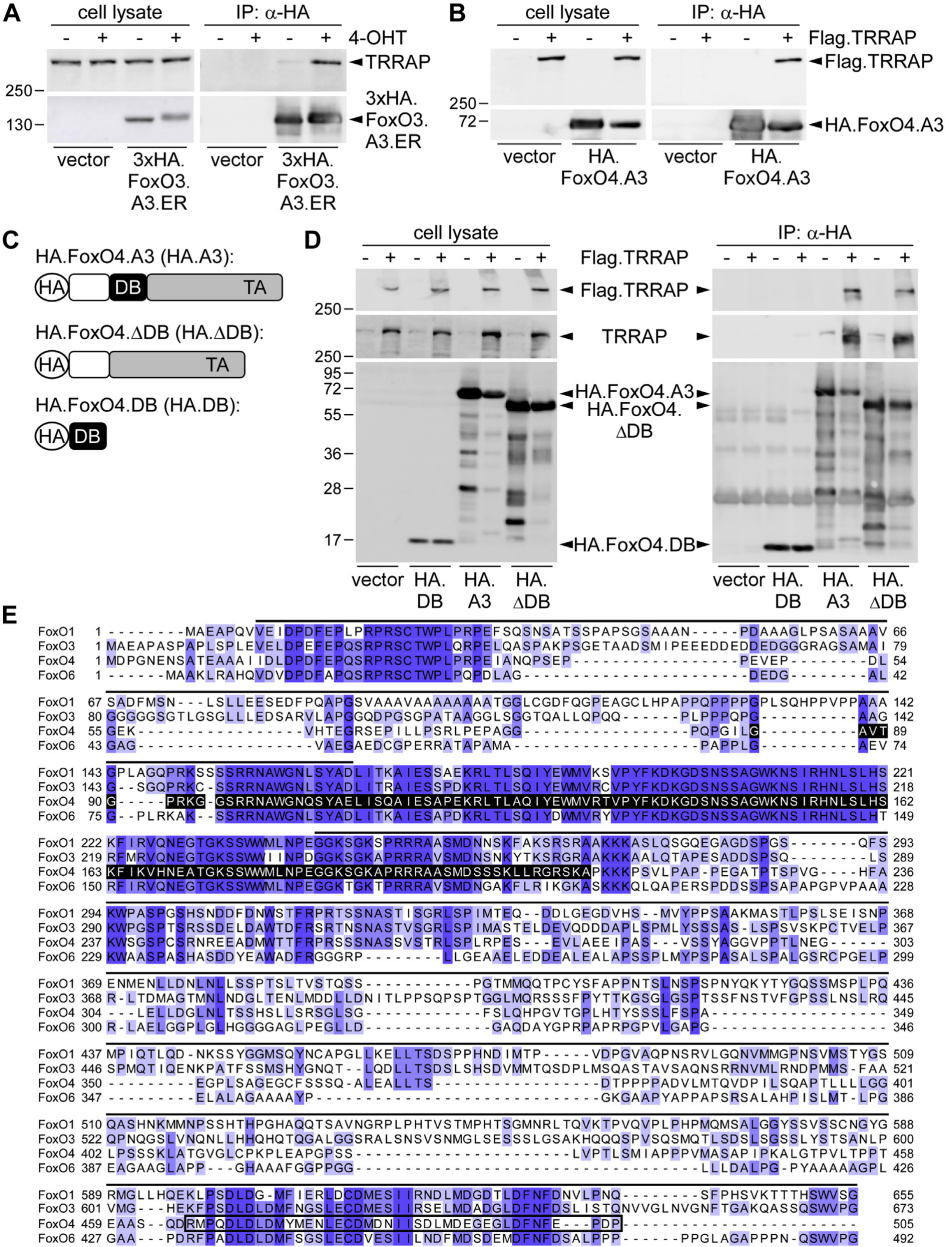
**FoxO–TRRAP interaction is conserved within different cell types and FoxO family members.***A*, Western blots, representing total lysates and α-HA IPs from HEK293 cells stably expressing an empty vector or 3xHA.HA.FoxO3.A3.ER and incubated for 16 h with culture medium (−) or 4-OHT (+). *B*, immunoblots of total lysates or α-HA IP fractions from HEK293 cells transfected with the indicated combinations of FLAG.TRRAP (+), HA.FoxO4.A3, and the respective empty vectors (−). *C*, schematic representation of the employed FoxO4 constructs. *D*, immunoblots of total lysates (*left*) and α-HA IPs (*right*) of HEK293 cells transiently transfected with HA.FoxO4.A3, HA.FoxO4.DB, or HA.FoxO4.ΔDB in combination with FLAG.TRRAP (+) or its empty vector (−), respectively. *E*, sequence alignment of the indicated human FoxO amino acid sequences. Protein sequences of human FoxO1 (knowledge base no.: Q12778), FoxO3 (knowledge base no.: O43524), FoxO4 (knowledge base no.: P98177), and FoxO6 (knowledge base no.: A8MYZ6) were obtained from the UniProt database (https://www.uniprot.org/) and aligned with the Jalview program, using the Clustal alignment algorithm. *Blue shades* indicate the degree of sequence conservation; the *black shaded area* corresponds to the sequence retained in the FoxO4.DB mutant; the *black line* on top of the alignment marks the FoxO4.ΔDB sequence; the FoxO4 TA is indicated by a *black transparent box*. 4-OHT, 4-hydroxytamoxifen; DB, DNA-binding domain; ER, estrogen receptor; FoxO3, forkhead box subclass O3; HA, human influenza hemaglutinnin; HEK293, human embryonic kidney 293 cell line; IP, immunoprecipitation; TA, transactivation domain; TRRAP, transformation/transcription domain–associated protein.

Experiments in human embryonic kidney 293 (HEK293) cells validated that TRRAP–FoxO3 interaction was conserved as we could also co-IP endogenous TRRAP from 4-OHT-treated HEK293 cells transfected with 3xHA.FoxO3.A3.ER (Fig. 2*A*). Importantly, TRRAP–FoxO3 interaction furthermore was independent of the ER fusion and also occurred with PKB phosphorylation–competent FoxO3 as IP of FLAG.TRRAP using an α-FLAG-specific antibody could likewise co-IP overexpressed WT HA.FoxO3 in HEK293, which contained functional PKB phosphorylation sites (Fig. S1). Notably, the capacity to interact with TRRAP in addition was conserved among FoxOs since FLAG-tagged TRRAP also readily coimmunoprecipitated with coexpressed A3 variants of FoxO1 or FoxO4 (Fig. 2*B* and data not shown). Hence, TRRAP–FoxO binding was neither EC specific or FoxO3 specific nor an artifact of a potential interaction of TRRAP with the introduced ER tag.

Next, with the help of two FoxO4 deletion mutants (illustrated in Fig. 2*C* and described in (10)), we narrowed down the region of interaction. Surprisingly, the highly conserved DNA-binding Fox turned out to be dispensable for interaction, as TRRAP binding was preserved when a FoxO4 variant lacking the DB (HA.FoxO4.ΔDB) was coexpressed (Fig. 2*D*). Consistently, an HA-tagged FoxO4 variant mainly encompassing the DB of FoxO4 (amino acids 98–183 (2); HA.FoxO4.DB) basically failed to interact with FLAG.TRRAP or endogenous TRRAP in α-HA co-IP experiments (Fig. 2*D*). Alignment of the protein sequences of all human FoxO family members revealed that the conserved regions in FoxO4 remaining as candidates for TRRAP interaction mainly comprised the ultimate N-terminal and the C-terminal transactivation domain (TA) (Fig. 2*E*).

### TRRAP is required for FoxO3 transactivation and modulates FoxO3 functions in ECs

**Figure 3:**
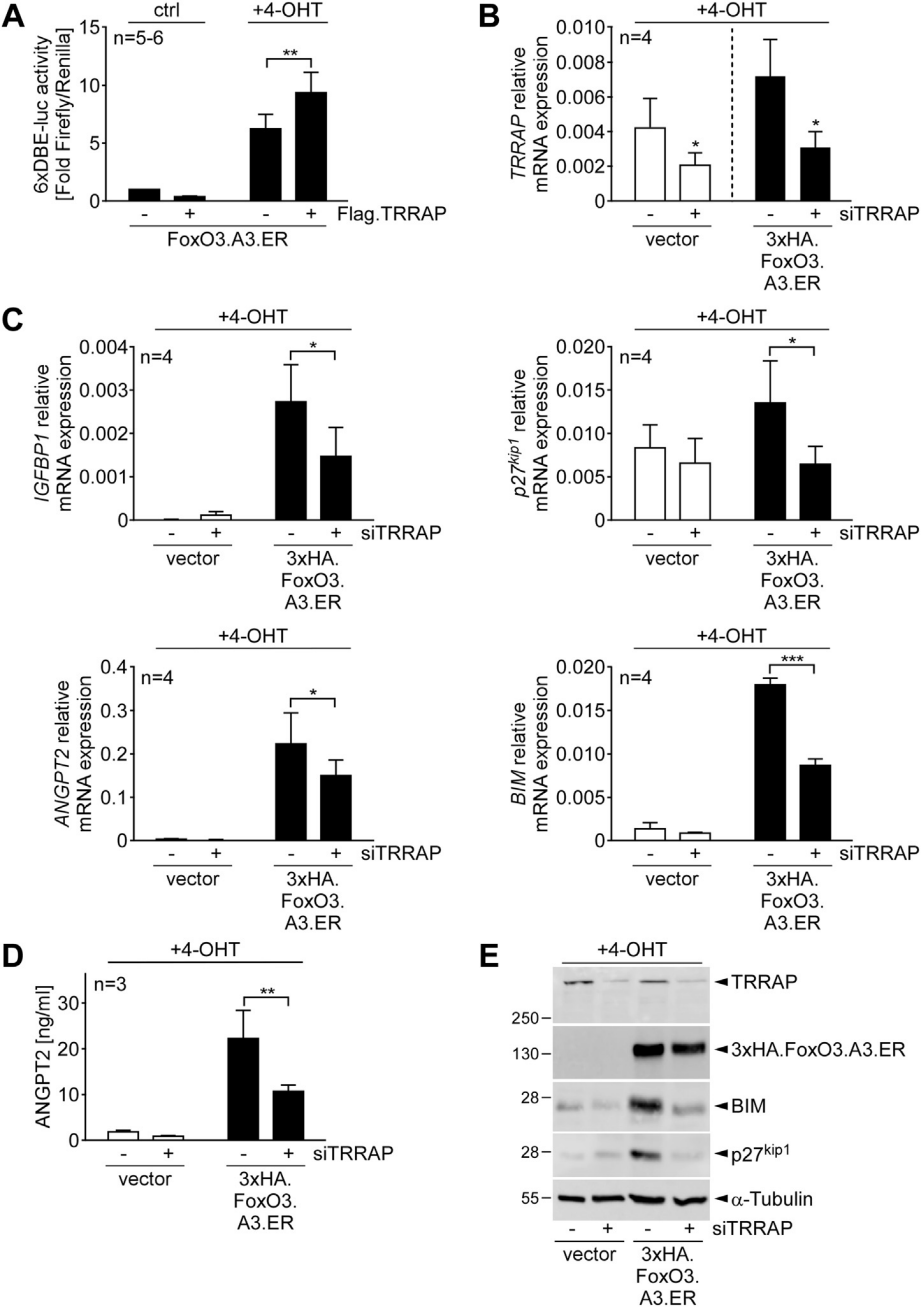
**TRRAP augments FoxO3 transactivation.***A*, luciferase assay, depicting 6×DBE-luc reporter activity in HUVECs expressing the indicated combinations of FoxO3.A3.ER with FLAG.TRRAP (+) or the respective empty vector (−) 16 h after incubation with 4-OHT or culture medium (ctrl). Ratios of luciferase activities obtained with the 6×DBE-luc reporter and the cotransfected constitutively expressed *Renilla* luciferase reporter (used to normalize for transfection efficiency) are displayed as fold activity of the experimental control. The bar diagram shows means + SD of n = 5–6 independent experiments performed in triplicate. *B* and *C*, RT–quantitative PCR analysis, showing relative mRNA expression of the indicated genes in HUVECs that were retrovirally transduced with empty vector or 3xHA.FoxO3.A3.ER and subsequently transfected with a scrambled siRNA (siSCR) (−) or a pool of two siRNAs against TRRAP (+). Combinations were each treated for 16 h with 4-OHT to induce FoxO3-dependent gene expression. The bar diagrams show relative GAPDH-normalized mRNA expression + SD of the indicated genes from n = 4 independent experiments. *D*, mean levels of ANGPT2 protein secretion into the culture medium as measured by ELISA. Depicted are mean ANGPT2 protein concentrations + SD measured in supernatants of n = 3 independent experiments performed with HUVEC that expressed an empty vector or 3xHA.FoxO3.A3.ER and were in addition transfected with siSCR (−) or a pool of two siRNAs against TRRAP (+). Cells were stimulated for 24 h with 4-OHT before harvesting the supernatants. *E*, immunoblot of total cell lysates from HUVECs treated as in (*D*). Statistical significances to the respective experimental control or between the indicated conditions in (*A*–*D*) are marked by *asterisks* (∗∗∗*p* ≤ 0.001; ∗∗*p* ≤ 0.01; ∗*p* ≤ 0.05; unpaired *t* test [*A* and *B*] or two-ANOVA with Sidak multiplicity correction [*C* and *D*]). 4-OHT, 4-hydroxytamoxifen; ANGPT2, angiopoietin 2; DBE, DAF-16 binding element; ER, estrogen receptor; FoxO3, forkhead box subclass O3; HA, human influenza hemaglutinnin; HUVEC, human umbilical vein endothelial cell; TRRAP, transformation/transcription domain–associated protein.

Because one of the candidate regions for TRRAP binding comprised the C-terminal TA domain, we next analyzed whether TRRAP overexpression may influence FoxO transactivation. To this end, we performed reporter assays using an established FoxO-responsive 6xDBE-luc reporter that consists of six tandem repeats of the consensus binding sequence for DAF-16 cloned in front of a *Firefly* luciferase reporter (26). Figure 3*A* illustrates that 4-OHT treatment expectedly resulted in a strong induction of the 6xDBE-luc reporter in HUVECs transfected with a combination of FoxO3.A3.ER and empty vector. Notably, additional FLAG.TRRAP coexpression further enhanced luciferase activity induced by conditional FoxO3.A3.ER activation, suggesting that TRRAP interaction promotes FoxO transactivation (Fig. 3*A*).

To further validate this hypothesis, we examined the influence of TRRAP knockdown on FoxO-dependent gene expression in ECs. For this, we infected HUVECs with either an empty retrovirus or a 3xHA.FoxO3.A3.ER–encoding retrovirus and tested the effect of transfecting a scrambled siRNA (siSCR) or two separately or pooled TRRAP-targeting siRNAs (siTRRAP) on FoxO3-mediated transcription. We first confirmed that transfection of either of the two single siRNAs alone as well as their transfection as combined pool efficiently reduced TRRAP protein (Fig. S2*A*). Next, pilot experiments revealed that transfection of each of the two siTRRAP independently reduced both mRNA expression of TRRAP and that of several selected FoxO3 target genes (15) (Fig. S2, *B* and *C*). Hence, all subsequent experiments were performed using a pool of those two siTRRAPs with which we typically achieved a ∼50% reduction of TRRAP mRNA levels (Fig. 3*B*). This translated into a significantly attenuated mRNA and protein induction of the selected FoxO target genes under 4-OHT, including IGFBP1, p27^kip1^, ANGPT2, and BIM (Fig. 3, *C*–*E*).

**Figure 4:**
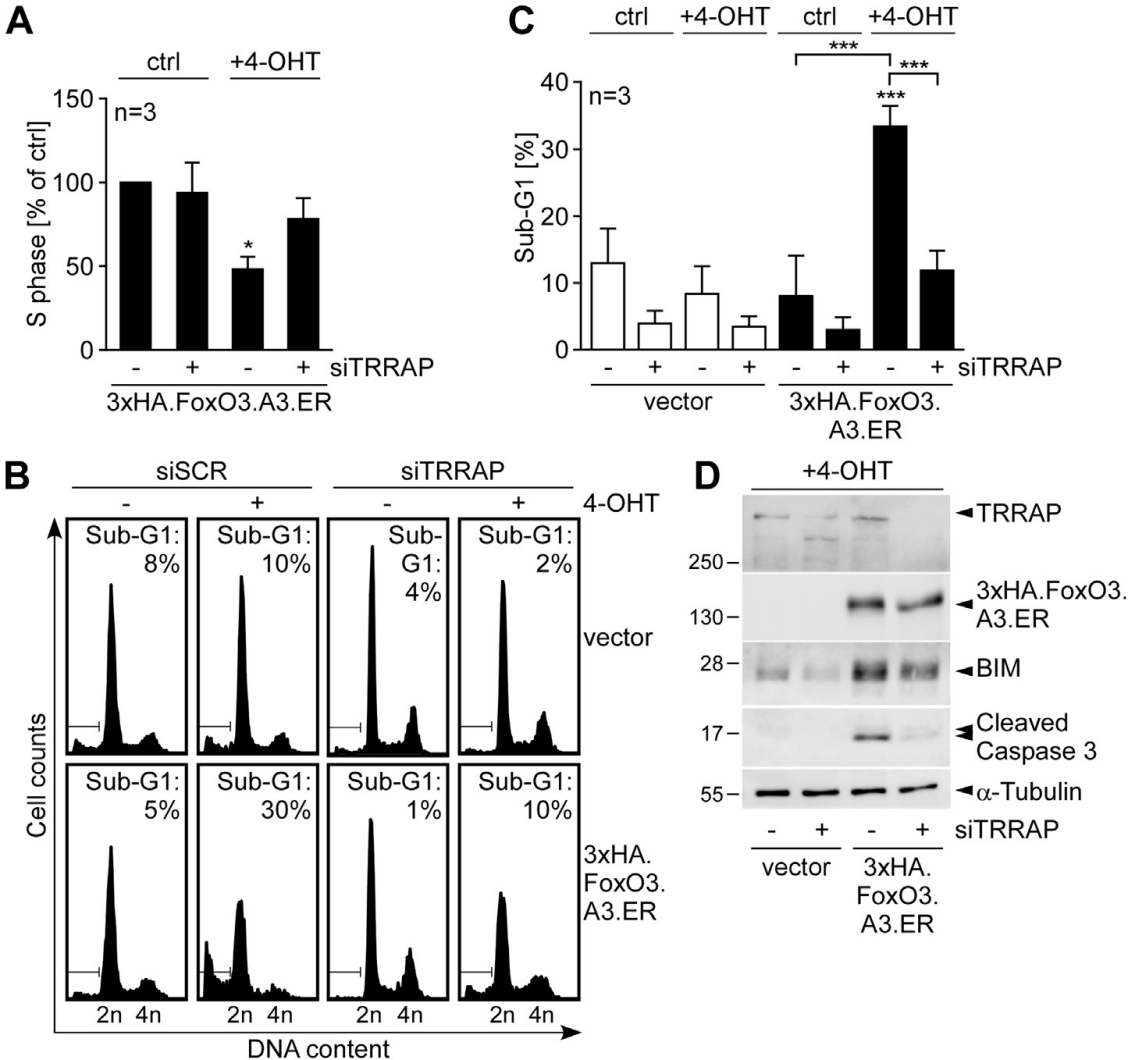
**TRRAP contributes to FoxO3-induced cell cycle arrest and apoptosis.***A*, histogram showing percentile S-phase content variation of HUVECs expressing 3xHA.FoxO3.A3.ER in response to transfection with scrambled siRNA (siSCR) (−) or of a pool of two siRNAs targeting TRRAP (+) and treatment with or without (ctrl) 4-OHT for 16 h. Data represent means of n = 3 independent experiments, each normalized to the S-phase content measured for the unstimulated siSCR/3xHA.FoxO3.A3.ER condition (arbitrarily set to 100%). Statistical differences in comparison to the normalized control are indicated by *asterisks* by (∗*p* ≤ 0.05; one-sample *t* test with Bonferroni–Holm multiplicity correction). *B*, representative DNA profiles of n = 3 independent experiments showing the impact of siSCR or siTRRAP transfection on 4-OHT-induced apoptosis of HUVECs transduced with empty vector or 3xHA.FoxO3.A3.ER. Cells were kept in culture with (+) or without (−) 4-OHT for 48 h. Indicated percentages represent the ratio of cells with sub-G1 content. *C*, quantification of apoptosis induction represented as mean percentage of subdiploidy + SD of n = 3 independent experiments performed as in (*B*). Statistical significance was determined by two-way ANOVA. Significant differences to the experimental control (siSCR/vector) are indicated by *asterisks* on top of the compared column (∗∗∗*p* ≤ 0.001). *D*, Western blots illustrating the efficiency of TRRAP knockdown, overexpression of 3xHA.FoxO3.A3.ER, and the expression of the apoptosis-related proteins BIM and cleaved (active) caspase 3. α-Tubulin served as loading control. Lysates of the differently siRNA-transfected HUVECs (siSCR: [−]; siTRRAPs: [+]) expressing 3xHA.FoxO3.A3.ER or the empty vector were taken 40 h after treatment with 4-OHT by pooling cells and culture supernatants prior to lysis to ensure inclusion of detached dying cells. 4-OHT, 4-hydroxytamoxifen; BIM, B-cell lymphoma 2–like protein 11; ER, estrogen receptor; FoxO3, forkhead box subclass O3; HA, human influenza hemaglutinnin; HUVEC, human umbilical vein endothelial cell; TRRAP, transformation/transcription domain–associated protein.

We previously reported that conditional FoxO3 activation in HUVEC triggers a rapid G1 cell cycle arrest followed by apoptosis, which critically requires BIM induction (15). Considering that TRRAP depletion strongly attenuated FoxO3-mediated expression of both p27^kip1^ and BIM, we next tested whether TRRAP knockdown might interfere with conditional induction of FoxO3-dependent G1–S cell cycle arrest or apoptosis. For this purpose, we carried out DNA profiling experiments with HUVECs, which were retrovirally infected with empty vector or 3xHA.FoxO3.A3.ER. These cells were exposed to 4-OHT for different times after siTRRAP or siSCR transfection. Expectedly, short-term 4-OHT treatment (16 h) of the siSCR/3xHA.FoxO3.A3.ER combination resulted in a clear G1–S cell cycle arrest of the cells with low apoptosis rates. This was evident by a small percentage of subdiploidy (indicating apoptotic cells), an increased G1 ratio, and a significant decrease of the S-phase population (Figs. S3 and 4*A*), which was not observed with the siSCR/vector combination (Fig. S3). By contrast, long-term 4-OHT treatment for 48 h resulted in a strong proapoptotic response characterized by an increased percentage of subdiploidy for the siSCR/3xHA.FoxO3.A3.ER-transduced HUVECs (Fig. 4, *B* and *C*). Intriguingly, siTRRAP depletion significantly antagonized both 4-OHT-dependent S-phase reduction (Figs. 4*A* and S3) and apoptosis levels in the 3xHA.FoxO3.A3.ER–infected cells (Fig. 4, *B* and *C*). The latter also correlated with a markedly reduced protein cleavage of the proapoptotic executioner caspase 3 and a less pronounced BIM protein induction upon conditional FoxO3 activation in the siTRRAP-cotransfected cells (Fig. 4*D*). Hence, TRRAP obviously not only modulates FoxO-dependent transcription but also influences FoxO-induced functional responses in ECs.

## Discussion

In an attempt to identify FoxO-interaction partners that may influence FoxO-dependent gene expression in ECs, we identified TRRAP as novel nuclear-binding factor of FoxO3. Our reporter assays clearly demonstrate that TRRAP overexpression promotes FoxO3-induced transactivation of FRE-dependent gene expression in ECs, suggesting that the TRRAP–FoxO interaction critically determines the transcription level of at least a subset of FoxO-dependent target genes. Accordingly, we were able to verify that TRRAP knockdown was capable of suppressing FoxO-induced upregulation of the established general FRE-containing targets IGFBP1 (13) and p27^kip1^ (10) and the endothelial-specific direct target gene ANGPT2 (15, 23). Although this further supports our conclusion that TRRAP binding serves to promote transactivation of FRE-dependent target genes in ECs, it is less clear how exactly TRRAP augments FoxO-dependent gene expression.

TRRAP is a large and highly conserved protein of the PIKK family, which further comprises DNA-dependent protein kinase, ataxia–telangiectasia mutated, ataxia- and Rad3-related, mammalian target of rapamycin, and suppressor of morphogenesis in genitalia (24). Unlike the other members of the PIKK family, TRRAP is catalytically inactive and does not act as a kinase. Rather, it functions as scaffold protein for several multiprotein transcriptional activation complexes containing histone acetyltransferase (HAT) function, whose composition varies in different cells and under different environmental conditions (24, 27). In humans, TRRAP particularly serves as a hub for recruitment of the SPT3–TAF–GCN5–acetylase (STAGA) and the Lysine acetyl transferase 5 (also known as TAT interacting protein 60 kDa [Tip60]) complexes, which recruit HAT enzymes belonging to the Gcn5-related *N*-acetyltransferases and the Moz, Ybf2/Sas3, Sas2, Tip60 (MYST) HAT families, respectively (28). Interestingly, in our MS analysis, we identified several other components of the Tip60 complex, such as E1A-binding protein p400 and DNA methyltransferase 1–associated protein 1 among the list of the top 30 enriched nuclear interaction partners (Table S1). This indicates that FoxO3 may particularly interact with the TRRAP–Tip60 complex in ECs. At this stage, we do not know if the recruitment of such TRRAP-associated HAT complexes may account for the enhanced transactivation of FoxO-dependent gene expression upon TRRAP overexpression. However, considering that TRRAP expression could enhance activity of a FoxO-responsive 6xDBE-luc reporter plasmid in transient transfection experiments, in which the reporter plasmid is unlikely to be packed into a chromatin-like structure, we exclude chromatin remodeling by TRRAP-associated HAT complexes as primary cause for its transactivating capacity. Nonetheless, HATs not only can acetylate core histones controlling the chromatin status but also can mediate acetylation of nonhistone substrates (29). Moreover, the aforementioned TRRAP-associated Tip60 and SPT3–TAF–GCN5–acetylase complexes promote interaction with several core factors of the basal transcription machinery, such as TATA box–binding protein and various TATA box–binding protein–associated factors, and facilitate loading of RNA polymerase II to the transcriptional complex (30). Thus, TRRAP might promote FoxO transactivation by assisting and augmenting interaction of FoxO3 with the basal transcription machinery.

Another major finding of our study was that TRRAP was critically required for BIM expression in ECs. This result is remarkable for two reasons. First, we previously identified BIM as an indirect FoxO target, whose expression could also be induced by a corresponding DNA-binding deficient FoxO3 point mutant (FoxO3.A3.H212R) in ECs. This mutant is unable to trigger activity of a 6xDBE-luc reporter (15), suggesting that TRRAP interaction also contributes to FoxO-dependent alternative gene regulation. Second, we could previously show that BIM induction critically contributes to FoxO3-dependent apoptosis (15), implying that the reduction of FoxO-induced apoptosis by TRRAP knockdown observed in our experiments was due to decreased BIM induction. These data indicate a key role of the TRRAP–FoxO interaction in the control of FoxO-dependent apoptosis. Such a notion predicts that the disruption of the TRRAP–FoxO interaction or the modulation of the composition of the TRRAP-associated transcriptional complexes might greatly influence the outcome of FoxO activation even within the same cell type. Remarkably, recent *in vivo* studies using a FoxO1.A3 transgene expressed in ECs in mice revealed that forced endothelial FoxO1 activation apparently did not induce an overt apoptotic response but resulted in vessel thinning and hypobranching (22). It is too early to speculate whether an altered TRRAP interaction or TRRAP complex composition under the experimental conditions *in vivo* may explain this result. Nonetheless, our finding that TRRAP knockdown could largely prevent FoxO3-induced apoptosis in ECs supports a scenario, in which FoxO-induced endothelial apoptosis might be subject to a context-dependent regulation at the level of TRRAP interaction. This may likewise apply to its antiproliferative effect and thus to its tumor suppressor function.

Of note, TRRAP was previously described to interact with several other transcription factors, including c-Myc, p53, and E2F transcription factors, which were shown to recruit TRRAP to their target promoters (31, 32). It is therefore well possible that FoxO3 recruitment to the promoters of its alternative targets occurs secondary to TRRAP interaction with another transcription factor that directs TRRAP to these sites allowing FoxOs to join the complex. Hence, TRRAP might not only function as a chromatin regulator but also as a platform, which coordinates the transcription of specific gene sets upon different stimuli. Following this perspective, such an organizational activity of TRRAP might explain why in different cell types or environments activation of FoxOs and other transcription factors triggers different outcomes. Such a concept may also well explain the observed induction of tissue-specific direct FoxO target genes such as ANGPT2, whose expression may depend on the accessibility of the FRE sites on its promoter at the chromatin level.

In summary, our data reveal TRRAP as a novel important regulator of FoxO-dependent gene expression in ECs, which crucially influences both direct and alternative gene expression in the endothelium and critically contributes to FoxO-induced cell cycle arrest and apoptosis in this cell type.

## Experimental procedures

All the presented results are representative of experiments independently reproduced at least three times, unless stated otherwise.

### Cell culture and stimulation

Primary HUVECs were purchased from PromoCell and cultured as described (15, 33). HEK293 cells and amphotropic Phoenix retrovirus producer cells were taken from the collection of the Department of Dermatology, University Hospital Würzburg and cultured in Dulbecco's modified Eagle's medium (Thermo Fisher Scientific) supplemented with 10% fetal bovine serum (Merck) and 100 μg/ml gentamycin (Sigma). All the cells were grown at 37 °C and with 5% CO_2_.

HUVECs overexpressing the FoxO3.A3.ER constructs or the empty vectors were stimulated with 100 nM 4-OHT (Calbiochem) for the indicated times to induce FoxO3 activity.

### Cloning and plasmids

To obtain the pBP-3xHA.FoxO3.A3.ER retroviral vector, pBP-FoxO3.A3.ER (15, 34) was amplified and linearized by PCR using the following primers: forward 5′-GCAGAGGCACCGGCTTCC-3′ and reverse 5′-GCCGGCGCCTAGAGAAGG-3’. By means of the HiFi DNA Assembly Protocol (New England BioLabs), a dsDNA fragment encoding a localization and purification tag (including 3xHA, GFP, and tobacco etch virus protease cutting site and an S-tag) (35) was inserted. The final expression vector for 3xHA.FoxO3.A3.ER protein was then generated by internal in-frame deletion of the GFP–tobacco etch virus–S-tag–encoding sequence by BamH1 digestion and subsequent religation of the plasmid.

The transient expression construct for FLAG-tagged TRRAP protein and the corresponding empty vector plasmid were a kind gift from Michael Cole, Geisel School for Medicine (Addgene plasmids: pCbS-FLAG [catalog no.: 32104] and pCbS–FLAG-TRRAP [catalog no.: 32103]) and have been described previously (32). Similarly, the pMT2-HA.FoxO4.A3, pMT2-HA.FoxO4.DB, pMT2-HA.FoxO4.ΔDB, pCDNA3-HA.FoxO3.A3.ER, pECE-HA.FoxO3 wt, and the 6xDBE-luc reporter constructs for transfections have been described previously (9, 10, 14, 26).

### Retroviral infections and transfections

Retroviral infections of HUVECs were performed in three consecutive rounds as described (15, 36). After 72 h of the third infection, positively transduced cells were selected adding puromycin (2 μg/ml; Applichem) to the medium for 16 to 18 h. Selected HUVECs were subsequently cultured in the absence of selection antibiotic for all experiments.

siRNA transfection of transduced HUVECs was performed after overnight puromycin selection using Oligofectamine Transfection Reagent (Thermo Fisher Scientific) based on the manufacturer’s protocol. The final concentration of total siRNA used for transfection was 200 nM. siRNAs targeting TRRAP were purchased: #1 from Thermo Fisher Scientific (catalog no.: 4427038; siRNA s15796) and #2 from Dharmacon/Horizon Discovery (catalog no.: D-005394-01) and used either separately or as pool of two siRNAs for all experiments. An siSCR (5‘-UUCUCCGAACGUGUCACGUdTdT-3’) was included as control. About 24 h after transfection, cells were reseeded for the experiments and processed as described.

HEK293 cells were transiently transfected in 10 cm dishes with 12 μg total DNA using a standard calcium phosphate protocol to express the different mutated proteins, and lysates were harvested 40 h after transfection.

### Cell lysis and fractionation

For Western blots and IPs, cells were lysed in E1A lysis buffer (150 mM NaCl, 50 mM Hepes [pH 7.5], and 5 mM EDTA), freshly supplemented with 20 mM β-glycerophosphate, 500 μM sodium orthovanadate, and 1× complete, EDTA-free protease inhibitor cocktail (Roche) as described (15).

To obtain nuclear and cytoplasmic extracts, cells were detached by trypsinization and pellets were washed two times with ice-cold PBS. Pellets were resuspended in 500 μl of buffer A (10 mM Hepes [pH 7.9], 10 mM KCl, 0.1 mM EGTA, 0.1 mM EDTA, freshly supplemented with 1 mM DTT, and 0.5 mM PMSF) and kept on ice for 15 min. Lysates were pressed 20 times through a 1 ml syringe with a 26G 3/8 (0.45 × 10) needle and centrifuged in a microcentrifuge for 5 min, 5000 rpm at 4 °C. Supernatants including the cytoplasmic extracts were harvested and stored on ice. Pellets containing the nuclear fraction were gently washed twice with buffer A. Extracted cell pellets were resuspended in 50 μl of buffer B (20 mM Hepes [pH 7.9], 0.4 M NaCl, 1 mM EGTA, 1 mM EDTA, freshly supplemented with 1 mM DTT, and 1 mM PMSF) and incubated for 15 min at 4 °C on a rocker to obtain the nuclear fractions. Cell debris was removed from both nuclear and cytoplasmic fractions by centrifugation for 10 min, 14,000 rpm, and 4 °C in a microcentrifuge, and supernatants were stored at −20 °C until use.

### Immunoassays

For immunofluorescence staining, retrovirally infected HUVECs were seeded at a density of 13,000 cells/cm^2^ on cover slips and one day after seeding stimulated for 16 h with 4-OHT. Cells were then fixed and permeabilized with methanol and stained with an α-ER antibody (catalog no.: sc-542 X; Santa Cruz) in 1% bovine serum albumin/1% normal goat serum/PBS buffer at a concentration of 1 μg/ml. A secondary goat–anti-rabbit antibody conjugated with Alexa568 (Thermo Fisher Scientific) at a concentration of 10 μg/ml in 1% bovine serum albumin/PBS buffer was used for fluorescence detection. Nuclei were stained with Hoechst 33342 (Sigma), and the cover slips were mounted using mounting medium from IBIDI. Images were taken using a fluorescence microscope equipped with a solid-state light source and a mounted monochrome digital camera (Nikon Ti), and pictures were analyzed using Nikon NIS-Elements AR 4.20.00 software.

For Western blot detection, equal amounts of protein lysates were mixed in a 1:4 ratio with 4× Läemmli buffer (250 mM Tris/HCl [pH 6.8], 40% glycerol, 10% β-mercaptoethanol, 8% SDS, and 0.1% bromophenol blue), boiled for 5 min at 95 °C, separated by SDS-PAGE, and blotted on nitrocellulose or polyvinylidene fluoride membranes. The following antibodies were used for detection: TRRAP (catalog no.: 3966), FoxO3a (catalog no.: 2497), and cleaved caspase 3 (catalog no.: 9664) were purchased from Cell Signaling, BIM (catalog no.: B7929) and α-tubulin (catalog no.: T5168) from Sigma, p27^kip1^ from BD Biosciences (catalog no.: 610241), FLAG (catalog no.: 600-401-383) from Rockland, and HA (catalog no.: 1867423) from Roche or (catalog no.: sc-805) from Santa Cruz.

After cell lysis with E1A lysis buffer as described previously, overexpressed HA-tagged FoxO proteins were immunoprecipitated using Pierce anti-HA magnetic beads (Thermo Fisher Scientific). For each sample, 25 μl (corresponding to 0.25 mg) of beads were equilibrated according to the manufacturer’s guidelines. A minimum of 600 μg of freshly harvested protein lysates were diluted to the same final volume and incubated with the beads for 2 h at 4 °C on an upside/down shaker. Three washing steps were performed, each with 300 μl of washing buffer (20 mM Hepes [pH 7.5], 300 mM potassium acetate, and 0.01% NP-40) for 5 min on an upside/down shaker at 4 °C. Elution was done at 70 °C on a rocker with 70 to 100 μl of 1× TruPAGE LDS sample buffer (Sigma) for the mass spectrometric analysis. For the other interaction experiments, proteins were eluted using 25 to 100 μl of 2× Läemmli buffer (125 mM Tris/HCl [pH 6.8], 20% glycerol, 5% β-mercaptoethanol, 4% SDS, and 0.05% bromphenol blue). FLAG.TRRAP was immunoprecipitated using anti-FLAG M2 Magnetic Beads (Sigma–Aldrich), according to the manufacturer's guidelines.

Release of ANGPT2 protein into the culture supernatant was quantified by ELISA using a commercial kit (Invitrogen; catalog no.: KHC1641) according to the manufacturer’s protocol. Culture supernatants were harvested 24 h after 4-OHT stimulation from HUVECs virally transduced with pBP-3xHA.FoxO3.A3.ER or the empty vector and subsequently transfected with siSCR or a pool of two siRNAs against TRRAP.

### Interactome analysis

HUVECs retrovirally infected with pBP-3xHA.FoxO3.A3.ER or the empty vector were stimulated for 16 h with 4-OHT, lysed with E1A lysis buffer, and proteins bound to 3xHA.FoxO3.A3.ER eluted by means of α-HA IP as described previously.

MS analysis was then performed from a single α-HA IP experiment. Briefly, protein precipitation was performed overnight at −20 °C with fourfold volume of acetone. Pellets were washed with acetone at −20 °C. Precipitated proteins were dissolved in NuPAGE LDS sample buffer (Life Technologies), reduced with 50 mM DTT at 70 °C for 10 min, and alkylated with 120 mM iodoacetamide at room temperature for 20 min. Separation was performed on NuPAGE Novex 4% to 12% Bis–Tris gels (Life Technologies) with Mops buffer according to the manufacturer's instructions. Gels were washed three times for 5 min with water and stained for 1 h with Simply Blue Safe Stain (Life Technologies). After washing with water for 1 h, each gel lane was cut into 15 slices.

The excised gel bands were destained with 30% acetonitrile in 0.1 M NH_4_HCO_3_ (pH 8), shrunk with 100% acetonitrile, and dried in a vacuum concentrator (Concentrator 5301; Eppendorf). Digests were performed with 0.1 μg trypsin per gel band overnight at 37 °C in 0.1 M NH_4_HCO_3_ (pH 8). After removing the supernatant, peptides were extracted from the gel slices with 5% formic acid, and extracted peptides were pooled with the supernatant.

Nano-liquid chromatography–tandem MS analyses were performed on an Orbitrap Fusion (Thermo Fisher Scientific) equipped with a PicoView Ion Source (New Objective) and coupled to an EASY-nLC 1000 (Thermo Fisher Scientific). Peptides were loaded on capillary columns (PicoFrit; 30 cm × 150 μm ID; New Objective) self-packed with ReproSil-Pur 120 C18-AQ, 1.9 μm (Dr Maisch) and separated with a 30 min linear gradient from 3% to 30% acetonitrile and 0.1% formic acid and a flow rate of 500 nl/min.

Both MS and MS/MS scans were acquired in the Orbitrap analyzer with a resolution of 60,000 for MS scans and 15,000 for MS/MS scans. Higher energy collisional dissociation fragmentation with 35% normalized collision energy was applied. A top speed data-dependent MS/MS method with a fixed cycle time of 3 s was used. Dynamic exclusion was applied with a repeat count of one and exclusion duration of 30 s; singly charged precursors were excluded from selection. Minimum signal threshold for precursor selection was set to 50,000. Predictive automatic gain control was used with automatic gain control with a target value of 2e5 for MS scans and 5e4 for MS/MS scans. EASY-IC was used for internal calibration.

Raw MS data files were analyzed with MaxQuant, version 1.6.2.2, an MS analysis package developed by the Max-Planck-Institute of Biochemistry (freely available at https://maxquant.org/) (37). Database search was performed with Andromeda, which is integrated in MaxQuant. The search was performed against the UniProt human database (Proteome ID: UP000005640; downloaded date: January 13, 2021; 97,795 entries [including all isoforms]) extended by the sequence of 3xHA.FoxO3.A3.ER. In addition, a database containing common contaminants was used. The search was performed with tryptic cleavage specificity with three allowed miscleavages. Protein identification was under control of the false discovery rate (<1% false discovery rate on protein and peptide-to-spectrum match levels). In addition to MaxQuant default settings (including 4.5 ppm main search peptide tolerance and 20 ppm MS/MS match tolerance), the search was performed against following variable modifications: Protein N-terminal acetylation, Gln to pyro-Glu formation (N-term. Gln), and oxidation (Met). Carbamidomethyl (Cys) was set as fixed modification. Label-free quantification (LFQ) intensities were used for protein quantification (38). Proteins with less than two identified razor/unique peptides were dismissed. Further data analysis was performed using R scripts developed in-house. Missing LFQ intensities in the control samples were imputed with values close to the baseline. Data imputation was performed with values from a standard normal distribution with a mean of the 5% quantile of the combined log10-transformed LFQ intensities and an SD of 0.1. For imputed proteins, the minimum number of razor/unique peptides was set to three. For the identification of significantly enriched proteins, boxplot outliers were identified in intensity bins of at least 300 proteins. Log2-transformed protein ratios of sample *versus* control with values outside a 1.5× (significance of 1) or 3× (significance of 2) interquartile range, respectively, were considered as significantly enriched.

### Luciferase assay

For luciferase assays, WT HUVECs were transfected in 10 cm dishes following the diethylaminoethyl-dextran protocol as previously described (15, 36). For all conditions, 1 μg of each overexpression plasmid, 4 μg of 6xDBE-luc reporter, and 266 ng of a commercial Renilla luciferase reporter expressed under control of a constitutive active promoter (Promega) were cotransfected. After one day of recovery, 18,000 cells/well were reseeded in triplicates into a 96-well plate, and after attachment, cells were stimulated for 16 h with 4-OHT. Cells were then processed for luminescence detection using the Dual-Glo luciferase assay system (Promega) according to the manufacturer’s protocol.

### RT–quantitative PCR

RNA from retrovirally infected HUVECs was isolated using a commercial RNA column purification kit (RNeasy MINI Kit; Qiagen), following the manufacturer’s indications, including the optional on-column genomic DNA digestion step. About 0.5 to 1 μg of purified RNA was then transcribed into complementary DNA using the QuantiTect Reverse Transcription Kit (Qiagen) according to the manufacturer’s suggestions. mRNA expression levels were assessed by quantitative real-time PCR using either TaqMan-based or SYBR Green–based assays and suitable master mix kits from Thermo Fisher Scientific. The following TaqMan probes were purchased from Thermo Fisher Scientific: ANGPT2 (catalog no.: Hs01048041_m1), BIM (catalog no.: Hs01076940_m1), GAPDH (catalog no.: Hs99999905_m1), p27^kip1^ (catalog no.: Hs01597588_m1), and TRRAP (catalog no.: Hs00268883_m1). For SYBR Green–based quantitative real-time PCR, the following primer pairs were used: IGFBP1 (forward: 5′-GGGACGCCATCAGTACC-3’; reverse: 5′-CCATTT TTTGATGTTGGTGAC-3′) and GAPDH (forward: 5′-CCACC CATGGCAAATTCC-3’; reverse: 5′-GATGGGATTTCCATT GATGACA-3′).

Ct values of the gene of interest were normalized against the Ct values of the housekeeping gene GAPDH (ΔCt), and the relative mRNA expression was calculated as 2^(−ΔCt)^.

### DNA profiles

Retrovirally infected HUVECs were transfected with siSCR or siTRRAP as described previously and incubated with 100 nM 4-OHT or medium for 16 h (early time point) or 48 h (late time point). Adherent cells and culture supernatants were pooled, washed twice with PBS, and fixed in ice-cold 70% ethanol for at least overnight. Fixed cell pellets were washed twice with PBS and then suspended in propidium iodide (PI) buffer (10 μg/ml PI and 250 μg/ml RNase in PBS) (15). PI intensity was then measured with a flow cytometer (BD Biosciences), and the DNA content of the cells together with the subdiploidy rates was quantified using FlowJo 7.6.5 (Tree Star Inc) software.

### Statistics

Statistical analysis was performed using the GraphPad Prism 6 biostatistical software (GraphPad Software, Inc). At least three independent experiments were averaged, and the SD was calculated to indicate error bars. Depending on the experimental setting, groups were compared by unpaired *t* test, one-column *t* test with Bonferroni–Holm correction, or two-way ANOVA followed by Sidak’s multiplicity testing to calculate multiplicity-adjusted *p* values. *p* values of <0.05 were considered significant.

## Data availability

The MS proteomics data have been deposited to the ProteomeXchange Consortium *via* the PRIDE partner repository (39) http://www.ebi.ac.uk/pride with the dataset identifier PXD027615.

## Supporting information

This article contains supporting information (15, 40, 41, 42).

## Conflict of interest

The authors declare that they have no conflicts of interest with the contents of this article.
