# Supplementary material for: Interaction of transcription factor FoxO3 with histone acetyltransferase complex subunit TRRAP modulates gene expression and apoptosis
Source: J Biol Chem. 2022 Feb 11;298(3):101714. doi: 10.1016/j.jbc.2022.101714 (PMC8914384; doi:10.1016/j.jbc.2022.101714)
Supplement: Supplemental Figures S1–S3 and Table S1 Legend [file mmc1.pdf]

Supporting information

Interaction of transcription factor FoxO3 with histone acetyltransferase complex subunit TRRAP Modulates Gene Expression and Apoptosis

Lorenza Fusi[1], Rupesh Paudel[1], Katharina Meder[1], Andreas Schlosser[2], David Schrama[1], Matthias Goebeler[1], and Marc Schmidt[1]

[1]Department of Dermatology, Venereology and Allergology, University Hospital Würzburg, Würzburg, Germany

[2]Rudolf Virchow Center, Center for Integrative and Translational Bioimaging, University of Würzburg, Würzburg Germany

Contents:

- Supplementary Figures 1 to 3

- Supplementary Table 1

- Supplementary References

Supplementary Figures

Figure S1: Interaction between TRRAP and FoxO3 wild type in HEK293 cells.
Western Blot, displaying total cell lysates and α-Flag IPs performed in HEK293 cells after transfection with Flag.TRRAP and HA.FoxO3 (+) or the combination of the empty vectors (-). The band indicated with * is non-specific and detected throughout all the samples by the applied α-HA antibody.

Figure S2: Comparison of different siRNA against TRRAP and their combination.
A) Representative Western Blot of total cell lysates taken from HUVEC transduced with an empty retrovirus (vector) or a retrovirus encoding 3xHA.FoxO3.A3.ER and subsequently transfected with a scrambled siRNA (-), or the indicated TRRAP siRNA alone (+) or as pool (++). All samples were harvested after 24 h treatment with 4-OHT.

B-C) Histograms, showing RT-qPCR analysis of relative mRNA expression of the indicated genes in HUVEC after incubation with 4-OHT for 16 h. Cells were retrovirally transduced with empty vector or 3xHA.FoxO3.A3.ER and then transfected with a scrambled siRNA (-) or two different siRNAs against TRRAP (+). Means of relative expression + s.d. of TRRAP (B) and FoxO-target genes (C) from two independent initial experiments are shown.

Figure S3: TRRAP knockdown interferes with FoxO3-induced G1-S cell cycle arrest in ECs.
DNA profiles and quantification of percentile cell cycle distribution of a representative experiment out of n=3, conducted with HUVEC, which were retrovirally infected with an empty vector or 3xHA.FoxO3.A3.ER and subsequently transfected with scrambled siRNA (-) or a pool of two siRNAs directed against *TRRAP* (+). Cells were incubated with or without (ctrl) 4-OHT for 16 h, to allow for proper cell cycle analysis without disturbing effects of massive apoptosis induction.

Supplementary Table

Table S1: List of nuclear FoxO3 interaction partners in HUVEC.
The table lists the identified and quantified proteins resulting from a single nanoLC-MS/MS analysis of the nuclear FoxO3 interactome (as described in the Experimental procedures part). Light yellow shading highlights the top 30 enriched proteins; light blue shading emphasizes the gene names corresponding to the identified proteins; green shadings marks data used to create Figure 1B in addition to information in the column “Normalized label free quantification (LFQ) ratio 3xHA.FoxO3.A3.ER+4-OHT_vs_vector+4-OHT”. The latter indicates the normalized log 2 of the ratios of the LFQ intensities of each detected protein in the 4-OHT-treated 3xHA.FoxO3.A3.ER condition versus the 4-OHT-stimulated empty vector sample. This value represents the enrichment of each protein in the sample with 3xHA.FoxO3.A3.ER overexpression, when compared with the empty vector. Proteins previously described as TRRAP binding proteins (40-42) among the top 30 enriched nuclear FoxO3 interaction partners are marked in bold. Obvious FoxO3 target genes such as *ANGPT2* (15) present among the top 30 enriched proteins were considered as potential false positives and thus were excluded from the yellow highlighting.

Supplementary References:

15. Czymai, T., Viemann, D., Sticht, C., Molema, G., Goebeler, M., and Schmidt, M. (2010) FOXO3 modulates endothelial gene expression and function by classical and alternative mechanisms. *J Biol Chem* **285**, 10163-10178

40. Fuchs, M., Gerber, J., Drapkin, R., Sif, S., Ikura, T., Ogryzko, V., Lane, W. S., Nakatani, Y., and Livingston, D. M. (2001) The p400 complex is an essential E1A transformation target. *Cell* **106**, 297-307

41. Finkbeiner, M. G., Sawan, C., Ouzounova, M., Murr, R., and Herceg, Z. (2008) HAT cofactor TRRAP mediates beta-catenin ubiquitination on the chromatin and the regulation of the canonical Wnt pathway. *Cell Cycle* **7**, 3908-3914

42. Doyon, Y., Selleck, W., Lane, W. S., Tan, S., and Cote, J. (2004) Structural and functional conservation of the NuA4 histone acetyltransferase complex from yeast to humans. *Mol Cell Biol* **24**, 1884-1896

Figure S1

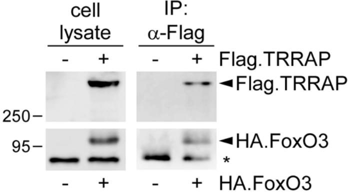

* unspecific band

Figure S2

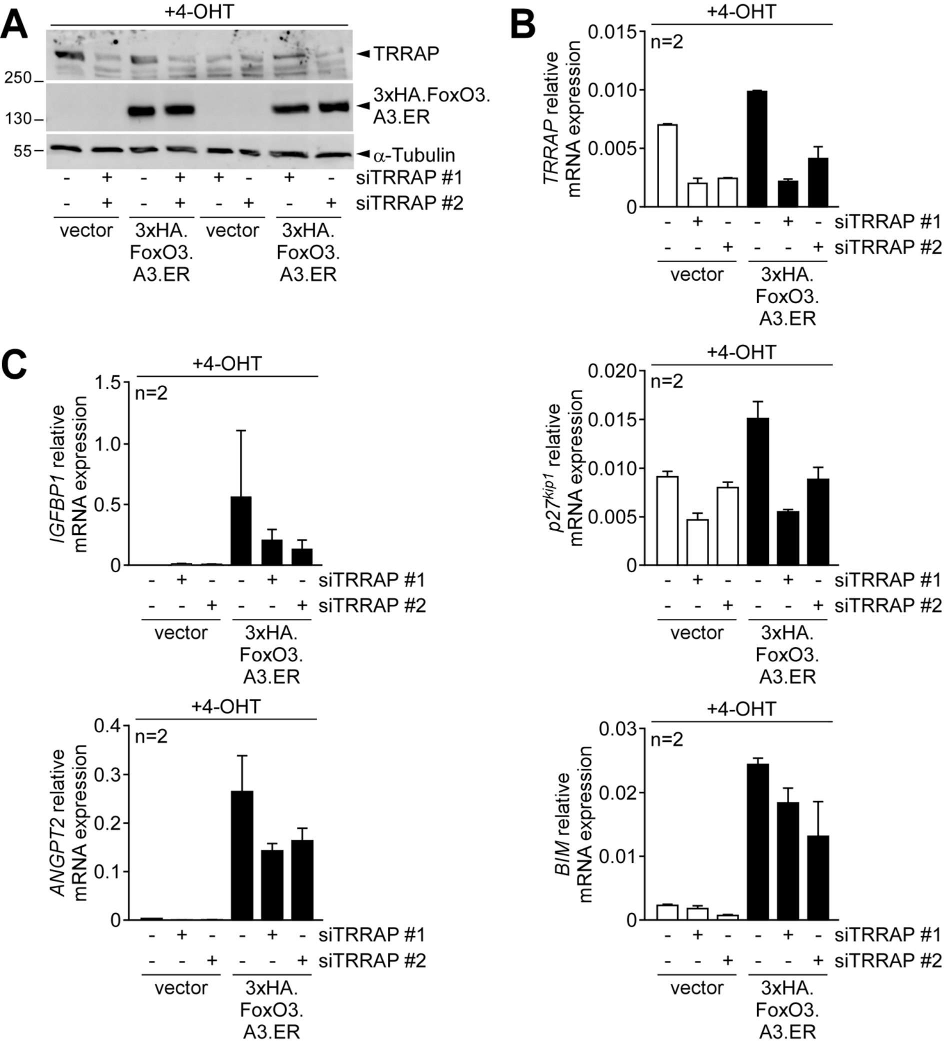
A
+4-OHT
TRRAP
3xHA.FoxO3.
A3.ER
α-Tubulin
250
130
55
siTRRAP #1
siTRRAP #2
vector
3xHA.
FoxO3.
A3.ER
B
+4-OHT
n=2
TRRAP relative
mRNA expression
0.015
0.010
0.005
0
C
IGFBP1 relative
mRNA expression
1.5
1.0
0.5
p27kip1 relative
mRNA expression
0.020
ANGPT2 relative
mRNA expression
0.4
0.3
0.2
0.1
BIM relative
mRNA expression
0.03
0.02
0.01

Figure S3

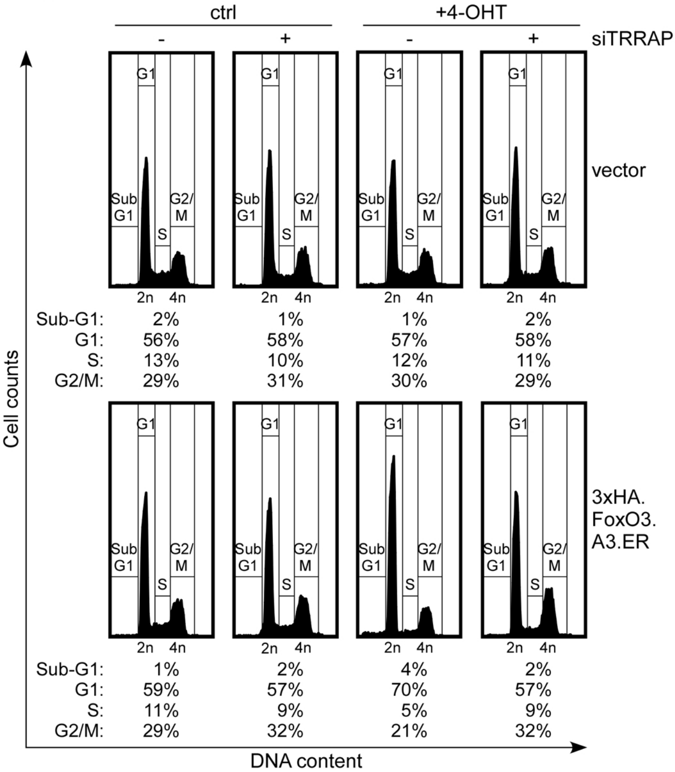
ctrl
+4-OHT
siTRRAP
-
+
-
+
vector
3xHA.
FoxO3.
A3.ER
G1
Sub
G1
S
G2/
M
2n
4n
Sub-G1: 2% 1% 1% 2%
G1: 56% 58% 57% 58%
S: 13% 10% 12% 11%
G2/M: 29% 31% 30% 29%
Sub-G1: 1% 2% 4% 2%
G1: 59% 57% 70% 57%
S: 11% 9% 5% 9%
G2/M: 29% 32% 21% 32%
Cell counts
DNA content
